# Molecular Antioxidants Maintain Synergistic Radical Scavenging Activity upon Co-Immobilization on Clay Nanoplatelets

**DOI:** 10.1021/acsbiomaterials.3c00909

**Published:** 2023-09-22

**Authors:** Adel Szerlauth, Szilárd Varga, Istvan Szilagyi

**Affiliations:** MTA-SZTE Lendület Biocolloids Research Group, Department of Physical Chemistry and Materials Science, Interdisciplinary Excellence Centre, University of Szeged, Szeged H-6720, Hungary

**Keywords:** antioxidant, synergism, gallic acid, nicotinamide adenine dinucleotide, layered double hydroxide

## Abstract

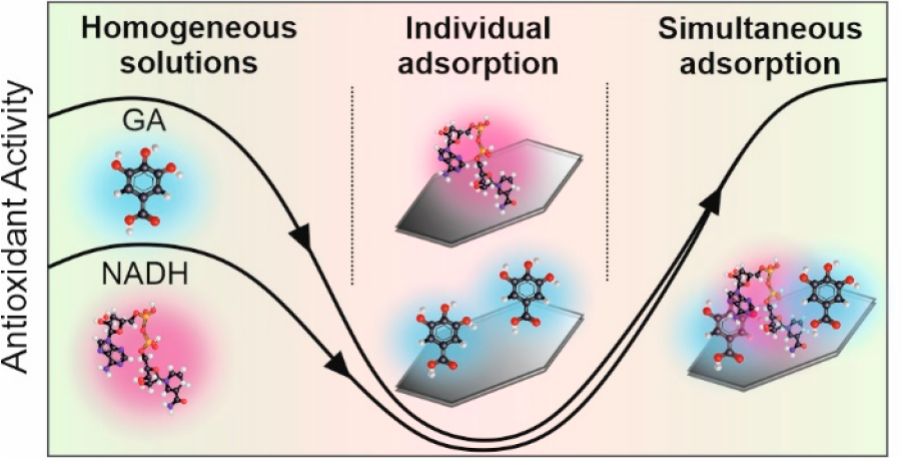

Unbalanced levels of reactive oxygen species (ROS) result in oxidative stress, affecting both biomedical and industrial processes. Antioxidants can prevent ROS overproduction and thus delay or inhibit their harmful effects. Herein, activities of two molecular antioxidants (gallic acid (GA), a well-known phenolic compound, and nicotinamide adenine dinucleotide (NADH), a vital biological cofactor) were tested individually and in combination to assess possible synergistic, additive, or antagonistic effects in free radical scavenging and in redox capacity assays. GA was a remarkable radical scavenger, and NADH exhibited moderate antioxidant activity, while their combination at different molar ratios led to a synergistic effect since the resulting activity was superior to the sum of the individual GA and NADH activities. Their coimmobilization was performed on the surface of delaminated layered double hydroxide clay nanoplatelets by electrostatic interactions, and the synergistic effect was maintained upon such a heterogenization of these molecular antioxidants. The coimmobilization of GA and NADH expands the range of their potential applications, in which separation of antioxidant additives is important during treatments or manufacturing processes.

## Introduction

Reactive oxygen species (ROS) are generated in normal biological processes, but environmental impacts (e.g., UV irradiation, air pollution, or food additives) can enlarge their production.^1,2^ Under normal physiological conditions, they are balanced by antioxidants. However, disruption of this balance causes oxidative stress, which can be responsible for many serious diseases (e.g., chronic inflammation or cancer).^3−5^ Antioxidants can delay or inhibit the negative consequences of increased levels of ROS by reducing them, forming metal chelates, or by interrupting free radical chain reactions.^6^ In addition to biomedical uses,^7−9^ there has been a great interest in the application of natural antioxidants in various industrial fields. It is a constant goal to find a solution to extend the shelf life of food products using antioxidants as preservatives or by designing food packaging materials of ROS scavenging activity.^10−13^ Antioxidants are also important ingredients of the modern cosmetic products, in which they are applied as anti-inflammatory or antimicrobial agents.^14,15^ Furthermore, they are useful as polymer additives to prevent degradation^16^ or to endow the polymer matrix with antioxidant features.^17,18^

The mechanism of molecular antioxidants is well studied, they can exert their effect in several ways such as hydrogen atom transfer, proton-coupled electron transfer or single electron transfer.^19^ Their combination may result in additive, synergistic, or antagonistic interactions. Accordingly, if the activity of two antioxidants is simply sum up, the interaction is additive, and if it is higher than the sum of their individual activity, then a synergistic effect takes place, while in the case of lower activity, antagonism occurs. The correct mechanism behind synergism is still not fully understood and different scenarios were assumed.^20^ To identify the relationship in combined antioxidant features, most of the studies apply the isobologram method using combination index or simply comparing the joint activity with the theoretical sum of the individual effects.^21^

Among phenolic acids, gallic acid (GA) has superb antioxidant, anticarcinogenic, antiviral, antiallergic, and neuroprotective activities.^22^ Its combination with other antioxidant molecules was studied in the past decades; however, further investigations are still needed to clarify the origin of their interaction in terms of activity and molecular orientation.^23,24^ It was shown that the extent of the joint effect is highly dependent on the molar ratio applied, the structure of the reaction partner, and the test method used to evaluate the antioxidant activity. For instance, GA mixed with epicatechin or catechin gave rise to different results depending on the antioxidant test used. Antagonistic and synergistic effects were observed with epicatechin and catechin, respectively, in the ferric reducing antioxidant power assay. Nevertheless, the tendency in activities was different in scavenging probes of free radicals, i.e., with epicatechin, synergism was reported, while for catechin, the relationship was largely dependent on the molar ratio.^25^

Nicotinamide adenine dinucleotide (NADH) is a well-known coenzyme with a crucial role in several biological processes (e.g., energy metabolism, DNA repair or transcription).^26^ Due to its hydride ion transfer ability, considerable research activity has been devoted to its application in different catalytic processes.^27,28^ For instance, direct antioxidant activity was studied to some extent.^29−31^ However, there is a lack of comprehensive investigations on NADH combination with other antioxidant molecules, despite of the existence of promising application fields such as cancer treatment by ROS elimination and subsequent decrease in the amount of ATP, which is suspected to be responsible for the multidrug resistance phenotype of cancer cells.^32^

The biomedical and industrial application of natural antioxidants is hindered by their sensitivity to the environmental conditions (e.g., pH, temperature, or electrolytes) and/or limited water solubility, nevertheless, heterogenization using suitable support materials may overcome this drawback.^33−35^ Layered double hydroxides (LDHs) are anionic clays and widely applied as host materials, because of their advantageous properties such as tunable structure, biocompatibility, cost-effectiveness, and relatively easy preparation techniques.^36−40^ LDH structure can be derived from brucite (Mg(OH)_2_) by isomorphic substitution of the divalent metal ions with trivalent ones. The most generic formula of an LDH is [M^2+^_*x*_M^3+^_1–*x*_ (OH)_2_] [A^*m0*–^_*x*/*m*_·*n*H_2_O], where M^2+^ and M^3+^ are the di- and trivalent metal cations, respectively, while A^*m*–^ is the interlayer charge compensating anion.^41,42^ The intercalation of biomolecules among LDH layers may reduce the sensitivity of the antioxidants, while the ROS scavenging activity may remain unchanged. However, intercalation can be hindered due to the limited interlayer spacing and high charge density of the layers.^43^ Delaminated or exfoliated LDH particles retains the advantageous properties of the original lamellar structure and can be a potential solution for the above-mentioned challenge.^44^ Furthermore, unilamellar LDHs possess high specific surface area, leading to increased amount of the adsorbed molecules on the surface.^45^ They may also serve as initial hosts during recovery of the lamellar structure upon intercalation of guest molecules. For example, GA was intercalated among LDH layers by a reconstruction procedure and furthermore, the so-prepared composite exhibited excellent photostability without loss of antioxidant activity.^46^ Other studies focused more on the release kinetics of GA from the interlayer space and the results revealed that the release mechanism and the antioxidant activity are strongly correlated.^47,48^ In addition to intercalation, surface adsorption driven by electrostatic interactions is another important tool to immobilize antioxidants on the outer particle surface.^49−51^ In some cases, the composite containing the molecular antioxidant was more effective upon adsorption rather than intercalation.^50^

**Scheme 1:**
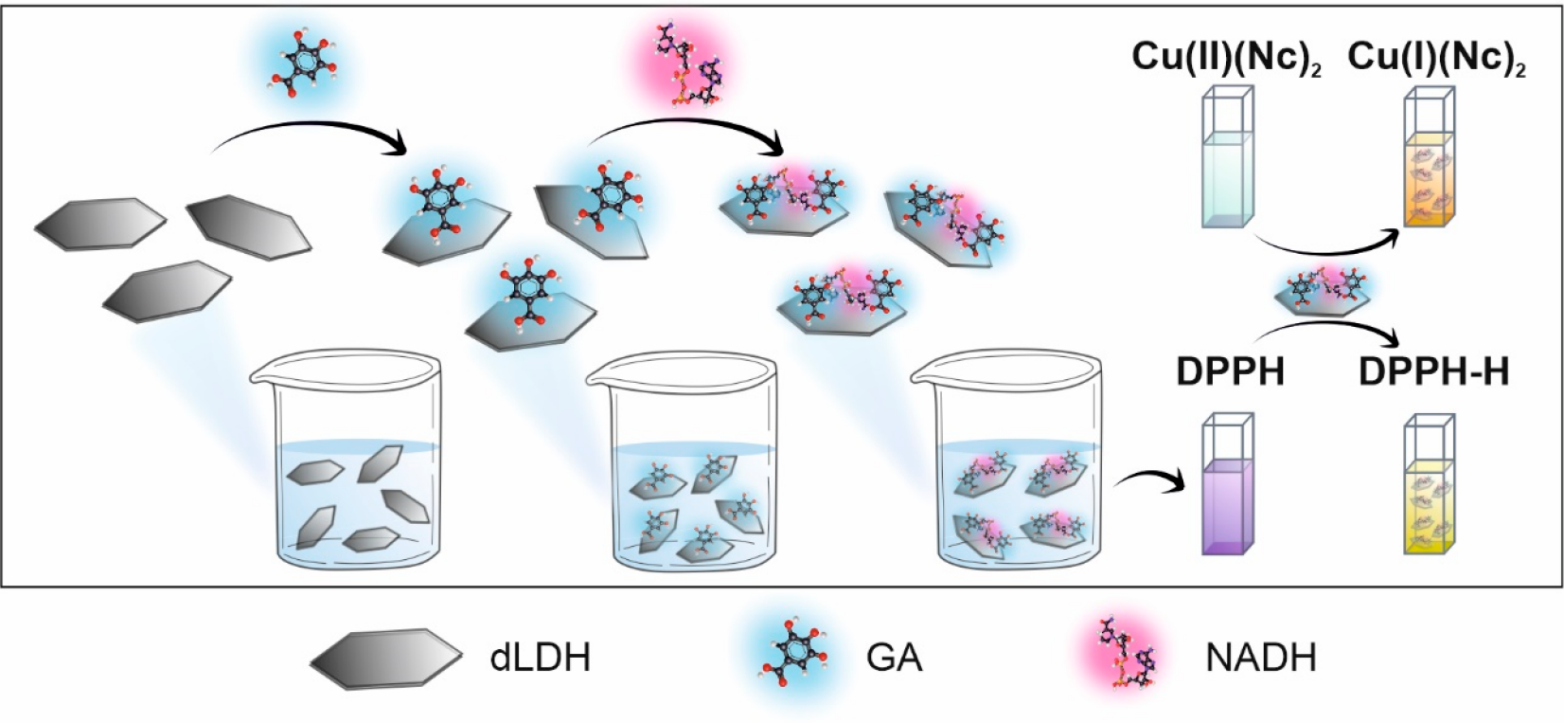
Schematic Representation of the Preparation and the Free Radical Scavenging Activity of dLDH/GA/NADH Composite

Herein, the antioxidant activity of GA, NADH, and their mixed solution was investigated by two well-known antioxidant activity assessment methods. The GA/NADH molar ratio was optimized to choose the optimal combination for further investigations, in which the molecules were coadsorbed on delaminated LDH (dLDH) nanoclay particles as confirmed in electrophoretic mobility measurements, while the colloidal stability was also probed. The synergism in antioxidant activity was systematically investigated in both homogeneous solution and dispersions of the heterogenized GA and NADH (Scheme 1).

## Experimental Section

### Materials

Magnesium nitrate hexahydrate (Mg(NO_3_)_2_·6H_2_O), aluminum nitrate nonahydrate (Al(NO_3_)_3_·9H_2_O), ammonium solution (23%), gallic acid (GA), sodium chloride (NaCl), sodium acetate (C_2_H_3_NaO_2_), 2,2-diphenyl-1-picrylhydrazyl (DPPH), ethanol (99%), copper chloride dihydrate (CuCl_2_·2H_2_O), neocuproine (Nc), and acetic acid (99%) were purchased from VWR in analytical grade. The reduced form of nicotinamide adenine dinucleotide (NADH) and the oxidized form of nicotinamide adenine dinucleotide (NAD) were obtained from Sigma-Aldrich and were analytical grade. Ultrapure water was produced with an Adrona B30 purification system.

### Preparation of Layered Double Hydroxide Nanosheets

Delaminated layered double hydroxide (dLDH) particles were prepared as described elsewhere.^52^ In brief, an aluminum nitrate (0.075 M) and magnesium nitrate (0.225 M) mixed salt solution as well as an ammonia solution (7 wt %) were added dropwise to a beaker, with simultaneous stirring. Then, the mixture was treated in an ultrasonic bath operating at 45 kHz frequency and 50 W power for 30 min. The sample was purified by washing and centrifugation steps, and the supernatant was used for further experiments. The detailed characterization and colloidal properties of dLDH particles was reported earlier.^52^

### Light Scattering Methods

A Litesizer 500 (Anton Paar) instrument was used for electrophoretic and dynamic light scattering measurements to determine the zeta potential and hydrodynamic radius (*R*_h_) data, respectively. The instrument is equipped with a laser (wavelength of 685 nm) operating at 40 mW power. The measurements were performed at 25 °C in backscatter mode (scattering angle of 175°). U-shaped Ω cuvettes (Anton Paar) were used for electrophoretic mobility measurements, while disposable plastic cuvettes (VWR International) for size determination. The relative error of the light scattering measurements is under 10%.

For electrophoretic measurements, samples containing 10 mg/L dLDH were mixed with the appropriate amounts of GA and NADH. Prior to measurements, the samples were allowed to rest overnight to achieve complete adsorption on the particle surface. The samples contained 1 mM NaCl as the background electrolyte. Zeta potentials (ζ) were calculated using the Smoluchowski equation:^53^
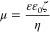
1where μ is the electrophoretic mobility, ε is the relative permittivity of the solvent, ε_0_ is the permittivity of vacuum, and η is the dynamic viscosity of the solvent.

The *R*_h_ of the particles was determined right after mixing the components. The final samples contained 10 mg/L dLDH and calculated amount of antioxidants. The *R*_h_ values were obtained with the Einstein-Stokes equation from the diffusion coefficient (*D*) determined by the cumulant fit on the intensity correlation function:^54^
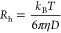
2where *k*_B_ is the Boltzmann constant and *T* is the absolute temperature.

### Free Radical Scavenging Activity

To evaluate the free radical scavenging activity, the DPPH assay was used.^55^ The DPPH solution was prepared in ethanol at a concentration of 46 mg/L. The reaction mixture was prepared by mixing 1 mL of DPPH solution and 1 mL of solutions containing GA and/or NADH in the desired concentrations, which ranged between 0 and 40 μM. The visible spectra were recorded using a Genesys 10S spectrophotometer, and the absorbance values were read at 517 nm, where the maximum absorbance of the DPPH solution is located. The reaction was allowed to run for 30 min. The remaining DPPH amount (DPPH %) was calculated by dividing the final absorbance value after the reaction between the DPPH and the antioxidants (*A*) with the initial absorbance of the DPPH solution (*A*_0_) prior to the reaction with any antioxidants:
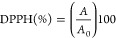
3The EC_50_ value, i.e., the antioxidant concentration responsible for scavenging half of the radicals in the solution, was calculated by plotting DPPH % against the antioxidant concentrations, followed by fitting the data with an appropriate mathematical function. For samples containing the composites, the particle concentration in the stock was 850 mg/L, GA concentration was 25 μM, while NADH was 62.5 μM for 0.4 and 16.7 μM for 1.5 molar ratios. To achieve the adsorption of the antioxidants on the dLDH surface, the dispersions were left overnight before the radical scavenging measurements. The reaction mixture here was prepared like the above-mentioned protocol, the only difference was the adding of 360 μL of 250 mM acetate buffer (pH 6) to the 1 mL of DPPH solution and the samples were completed to 2 mL with the antioxidant dispersions. Note that the dLDH dispersions alone did not absorb at the wavelength of interest and did not catalyze the DPPH reduction within the time frame of the assay. The relative error of the DPPH test is 5%.

To evaluate the optimal molar ratios, the decrease in DPPH (%) (ΔDPPH (%)) was calculated with the following equation:
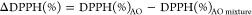
4The joint effect of the antioxidants was evaluated by calculating combination indices (CIs).^20^ To determine the CI values for each multicomponent system, the following equation was applied:
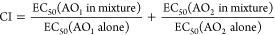
5For dLDH containing systems, EC_25_ (the antioxidant concentration responsible for the decomposition of 25% of DPPH radicals) was applied, because dLDH/GA and dLDH/NADH systems did not reach the 50% scavenging activity. Based on the CI values, the relationship between the antioxidants can be obtained. As described elsewhere, the interaction is synergistic, additive or antagonistic, if the CI < 1, CI = 1 or CI > 1, respectively.^56,57^

### Copper Reducing Antioxidant Capacity (CuPRAC) Assay

To explore redox activities, the CuPRAC assay was carried out.^58^ A copper-neocuproine (Cu(II)(Nc)_2_) complex solution was prepared by codissolving CuCl_2_ (0.01 M) and Nc (0.02 M) in a mixture of ethanol and water in a 1:4 volume ratio. The reaction mixture contained 250 μL Cu(II)(Nc)_2_ solution completed with the antioxidant stocks and water to 2050 μL. The concentration of the antioxidants was varied in the range of 0–50 μM. The change in absorbance was measured at 450 nm after 30 min reaction time, at the maximum absorbance of the Cu(I)(Nc)_2_ solution (formed form Cu(II)(Nc)_2_ upon reduction). Linear fit was performed on the data and trolox equivalent antioxidant capacities (TEAC) were calculated by dividing the slope determined for each antioxidant by the slope for trolox, which was used as the reference molecule:^33^
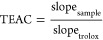
6The relative error of the CuPRAC assay is about 5%.

## Results and Discussion

### Synergistic Radical Scavenging Activity of GA and NADH

**Figure 1:**
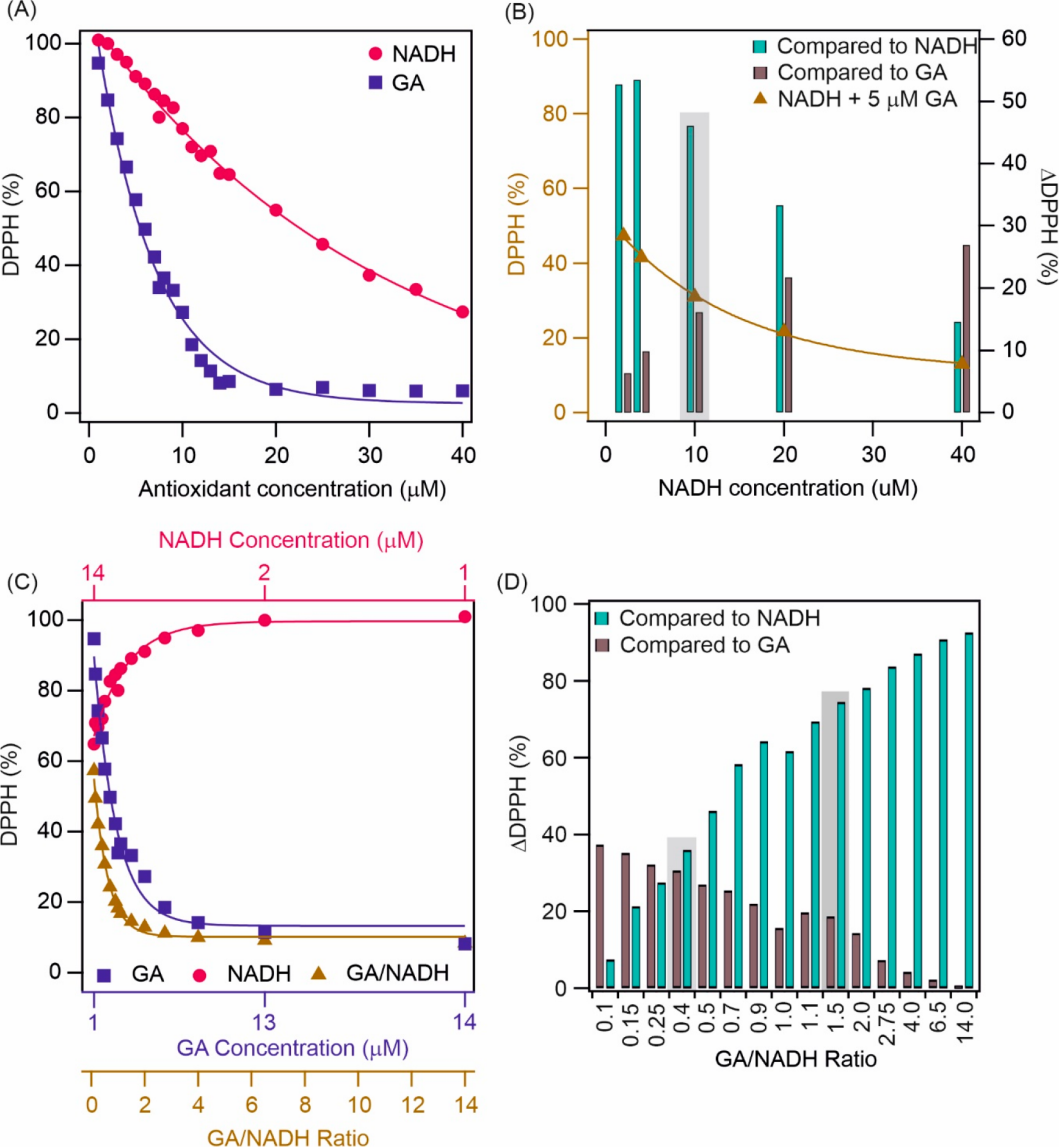
(A) Radical scavenging activity of GA and NADH. (B) The selected data for the DPPH (%) versus NADH concentration (yellow triangles) and the ΔDPPH (%) (colored bars) in samples containing 5 μM GA and various amounts of NADH. (C) The radical scavenging activity of the mixture was at 15 μM total antioxidant concentrations with different GA/NADH ratios. Note that for the better comparison, the individual antioxidant activity of NADH and GA is also labeled on the graph. (D) The ΔDPPH (%) at different molar ratios compared to the DPPH (%) for GA and NADH. The molar ratios chosen for the further measurements are labeled with a gray shadow.

First, the antioxidant activity of GA and NADH was investigated by using the DPPH assay. DPPH is a relatively stable radical with a purple color in ethanol. Upon reduction of the radical, the solution changed to yellow. This color change can be monitored at 517 nm, the absorption maximum of the DPPH radical. In Figure 1A, the DPPH (%), the percentage corresponding to the nonreacted DPPH, is plotted against the initial antioxidant concentration in the reaction mixture. As the concentration of the antioxidant increased, DPPH (%) decreased. Calculating the EC_50_ values (Table S1), GA has significantly lower EC_50_ values and thus higher free radical scavenging activity than NADH, which showed only moderate antioxidant activity under the experimental conditions applied.

To determine their joint radical scavenging activity, the concentration of GA was kept constant (5 μM), whereas the NADH concentration was systematically varied in the samples (Figure 1B). To analyze individual and mixed antioxidant activities, ΔDPPH (%) was calculated. Although the highest ΔDPPH (%) compared to NADH was detected at 7 and 9 μM of total antioxidant concentration, the ΔDPPH (%) compared to GA was negligible at these concentrations. At 15 μM total antioxidant concentration the ΔDPPH (%) compared to NADH remained ∼50%, in addition ΔDPPH (%) compared to GA also increased above 10%. Therefore, to determine the optimal ratio for further studies and to explore synergistic effects, the total antioxidant concentration was set to 15 μM and the molar ratio of GA and NADH was systematically varied (Figure 1C). The DPPH (%) was lower at all antioxidant ratios than that at the corresponding GA or NADH concentrations. At 0.4 GA/NADH ratio, the ΔDPPH (%) remained similar for both antioxidants. However, to investigate the system at a molar ratio higher than 1, a 1.5 molar ratio was also applied (Figure 1D). At this condition, the ΔDPPH (%) for NADH increased significantly, while for GA, it was around 20%. Thus, molar ratios of 0.4 (GA/NADH/0.4) and 1.5 (GA/NADH/1.5) were chosen for further measurements.

**Figure 2:**
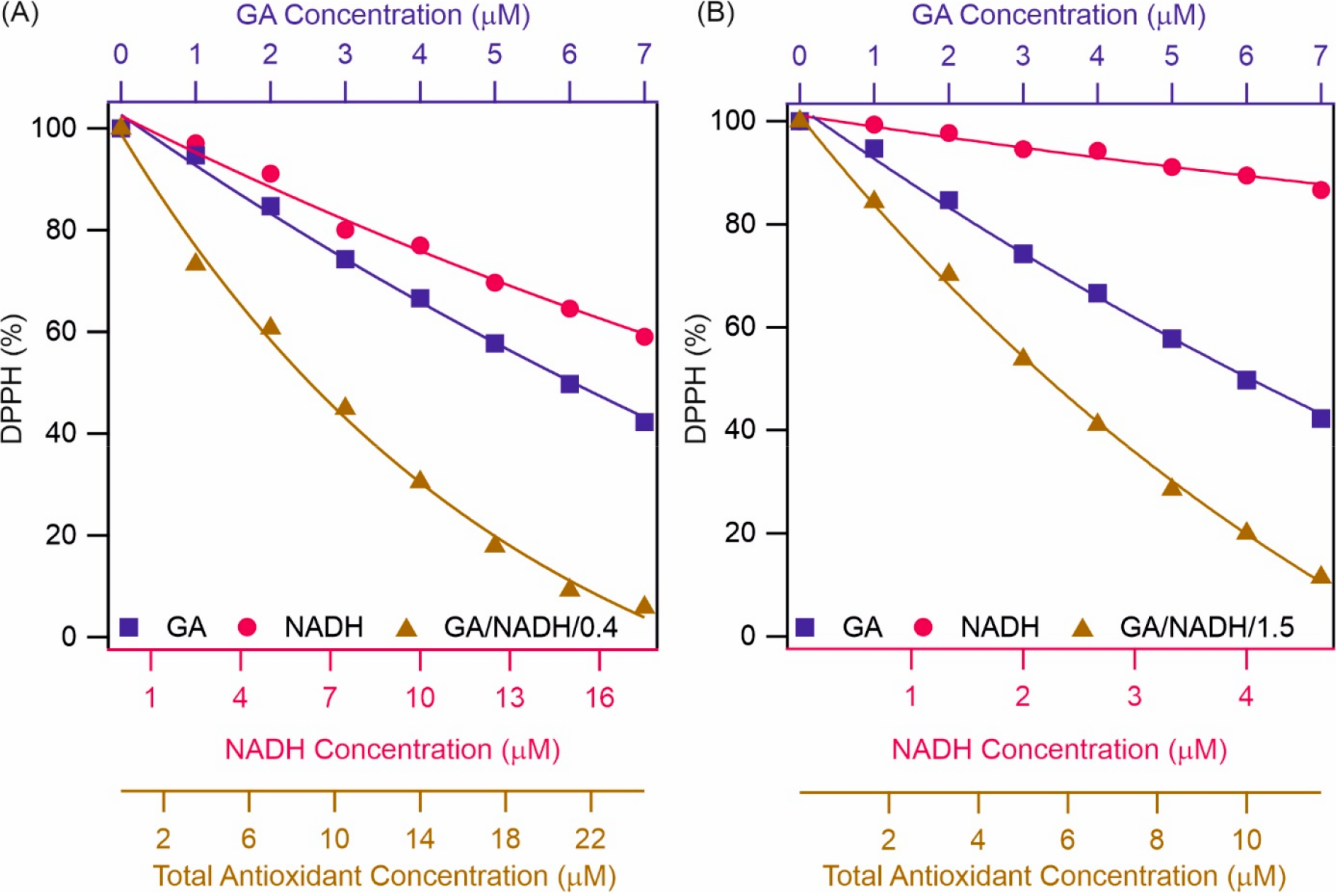
Radical scavenging activity of GA, NADH, and their mixture at (A) 0.4 and (B) 1.5 GA/NADH molar ratios. For easier comparison, DPPH (%) data for the individual GA and NADH systems are also shown. The lines are mathematical fits used to determine the EC_50_ values.

First, the GA/NADH/0.4 system was investigated (Figure 2A). A significant difference in antioxidant activity was observed between the sum of the activities of individual antioxidants and the ones measured for the mixture. The EC_50_ values given in Table S1 indicate that the mixture showed superior antioxidant activity in comparison to the sum of the individual GA and NADH. Accordingly, the EC_50_ of GA/NADH/0.4 mixture was found to be 8.8 μM, which contains 2.5 μM GA and 6.3 μM NADH, which is much lower than the sum of the EC_50_ of GA and NADH (28.6 μM), indicating the synergistic effect upon mixing the two molecular antioxidants. For GA/NADH/1.5 a similar effect was observed (Figure 2B). The mixture showed higher radical scavenging activity than the individual counterparts together. The EC_50_ value for GA/NADH/1.5 was determined to be 5.5 μM. Based on these results, the 1.5 molar ratio seemed to be a more effective combination, because of the lower EC_50_ value compared to the 0.4 molar ratio case.

Comparing the individual antioxidant doses, one can see that lower concentrations of GA and NADH are needed in the mixtures to achieve the same radical scavenging effect. The CIs were 0.70 and 0.65 for GA/NADH/0.4 and GA/NADH/1.5, respectively (Table S2). The CI values are less than 1 indicating a synergistic effect. Such a synergism may have originated from the electron transfer of NADH through oxidized GA to DPPH radicals. Some theories state that the weaker antioxidant can reduce the stronger one so that the latter can in turn interact with the free radicals present in solution.^20^ Here, it was assumed that GA, after reaction with a DPPH molecule, transfers electrons from NADH to the free radicals. To further prove this theory, the combined activities of GA and NAD (the oxidized form of NADH) was explored. NAD did not have remarkable antioxidant activity, and the combination with GA did not show significantly different effect from the activity of GA (Figure S1). The EC_50_ of the GA/NAD/0.4 system is 20.9 μM, which is composed of 6.0 μM GA and 14.9 μM NAD. This is in good agreement with the individual EC_50_ value of GA, so NAD did not contribute to the antioxidant activity. This further confirms the proposed mechanism above because unlike NADH, NAD has no transferable electron and hence, synergistic effect in DPPH scavenge cannot take place between GA and NAD.

### Immobilization of GA and NADH on dLDH Surface

**Figure 3:**
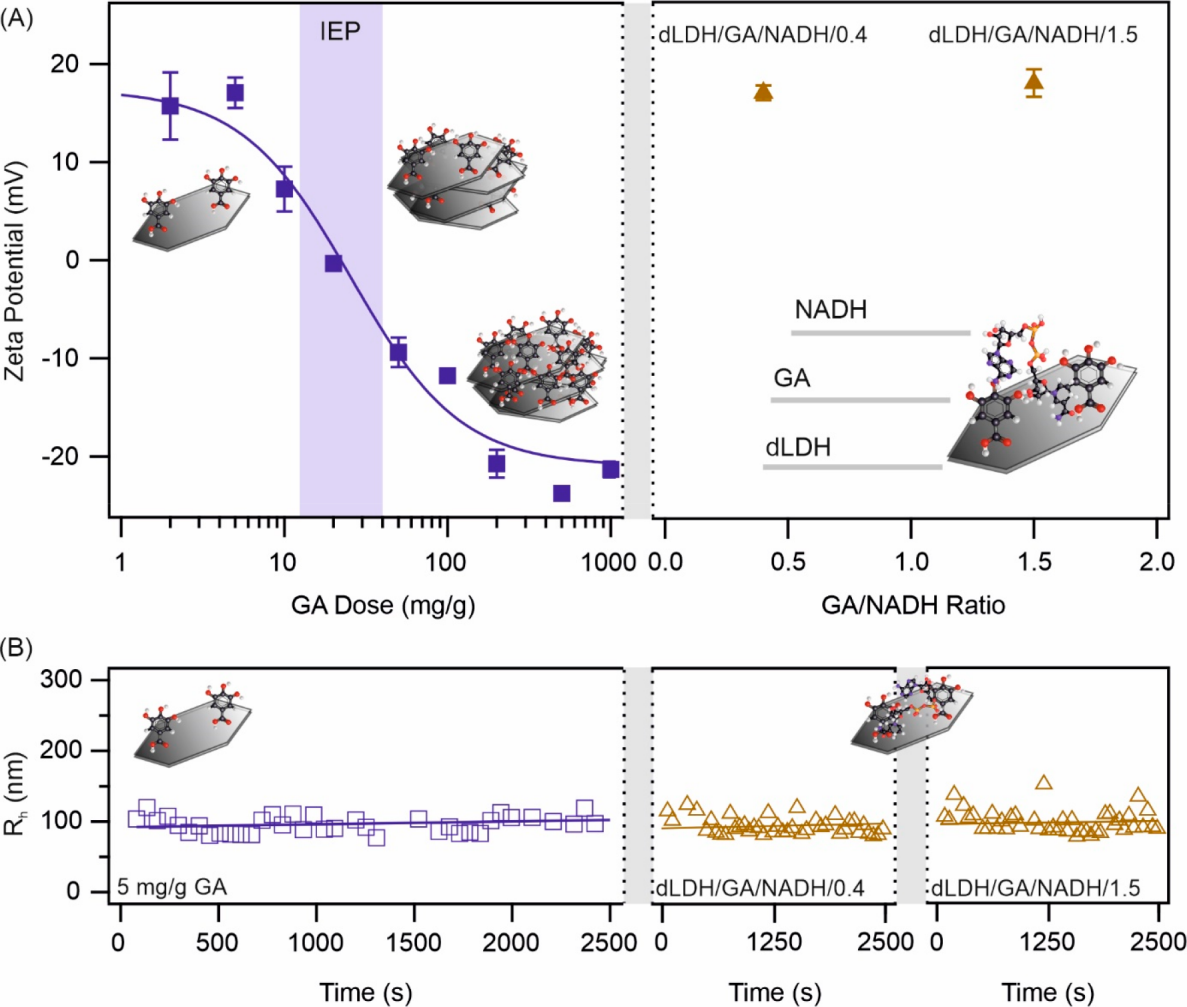
(A) Change in zeta potential of dLDH particles after immobilization of GA (left) and after simultaneous adsorption of GA and NADH at two molar ratios (right). (B) The hydrodynamic radius of dLDH versus time in the presence of GA (left) as well as of GA/NADH/0.4 and GA/NADH/1.5 (middle and right, respectively). In all experiments, 1 mM NaCl was applied as background electrolyte.

To widen the applicability of GA and NADH, they were heterogenized, i.e., adsorbed on the dLDH surface individually and together. To monitor the surface charge properties during adsorption of the antioxidant, electrophoretic light scattering measurements were performed to determine the zeta potential of the particles (Figure 3A). This parameter gives important information about the surface charges, which should significantly change upon adsorption of molecules on oppositely charged surfaces.^33,51,59^ At low GA doses, the initial potential of dLDH (∼20 mV) did not change significantly. With the increase in GA dose, the values decreased such that charge reversal occurred resulting in negatively charged particles at high GA concentrations. The isoelectric point, the GA dose, at which the particle charge is neutralized due to GA adsorption was observed at 20 mg/g. Furthermore, a smaller extent of NADH adsorption was indicated by the zeta potential data (Figure S2A). As a result, charge reversal took place to a much smaller extent. Accordingly, no significant change in dLDH charge was observed at low concentrations, and only slightly negatively charged particles were detected even at the highest NADH doses applied. Such a significant difference in the adsorption properties is due to the presence of carboxylate functionalities in the GA structure, whose affinity to the alkaline dLDH material is particularly high, as reported earlier.^60,61^

Tendencies in colloidal stability measurements showed good agreement with the zeta potential data considering interparticle forces of electrostatic origin.^59,61,62^ At low GA doses, the dispersions were stable, i.e., hydrodynamic radii remained constant (Figure 3B and Figure S2B). By increasing GA concentration, the dispersion turned to be unstable, fast aggregation occurred, where the hydrodynamic radii increased steeply. The highest increase in the radii was experienced near the isoelectric point. By further increasing the GA dose, the dispersion remains unstable, hydrodynamic radii increased rapidly, and fast aggregation occurred (Figure S2B). Based on the stability measurements 5 mg/g GA dose was applied in further studies, since the stable particle dispersions of high specific surface area must be applied during coadsorption of NADH. Similar trend was observed for individual NADH system (the inset of Figure S2A). At low doses, no change in hydrodynamic radius can be observed, while with further increase of the antioxidant dose, fast aggregation occurred. Considering the antioxidant activity and colloidal stability aspects, dLDH/NADH composites of a 50 mg/g dose were used in further investigations, where NADH was solely present in the materials in question.

Furthermore, based on the results of the light scattering measurements, 5 mg/g GA, and calculated NADH doses were applied to study the synergistic effects at 0.4 and 1.5 molar ratios, which were the most effective ones in the DPPH assays performed in homogeneous solutions. The final composites contained 5 mg/g (0.29 μM) of GA with 48 mg/g (0.72 μM) of NADH (dLDH/GA/NADH/0.4) and 5 mg/g (0.29 μM) of GA with 13 mg/g (0.19 μM) of NADH (dLDH/GA/NADH/1.5). The coadsorption of both molecules had no significant effect on the charge of the particles, i.e., the composites containing both GA and NADH remained positively charged (Figure 3A) under the experimental conditions investigated. Hydrodynamic radius data (Figure 3B) indicate that the particle size did not change significantly upon coimmobilization of GA and NADH, which trend also confirms the formation of stable dispersions of dLDH/GA/NADH/0.4 and dLDH/GA/NADH/1.5.

### Radical Scavenge by Heterogenized Antioxidants

Initially, the same protocol was applied to measure the radical scavenging activity as with the nonimmobilized compounds. Nevertheless, a striking difference was observed in the spectra once the immobilized antioxidants were present. Instead of the decrease in the absorbance at 517 nm, a new peak appeared at 420 nm (Figure S3). This new absorption band is related to the formation of DPPH^–^ ion, which can be formed in basic conditions by the deprotonation of the reduced form of DPPH. Therefore, as described in the Experimental Section, a modified DPPH scavenging protocol was applied with the immobilized antioxidants^63^ leading to the disappearance of the band corresponding to the DPPH^–^ molecule (Figure S4). The acidic condition (pH 6) set by the buffer solution provides the formation of DPPH-H, and prevents the deprotonation and thus the formation of the DPPH^–^ molecule.

For the sake of comparison, the radical scavenging activity of the free antioxidants was also measured with the modified DPPH assay at pH 6 (Figure S5). No significant change can be observed for GA, however, the NADH EC_50_ value increased remarkably indicating the decrease of radical scavenging activity of NADH at pH 6 (Table S1). Therefore, the EC_50_ value related to the GA/NADH/0.4 mixture also increased, as the radical scavenging activity decreased due to the change in the activity of NADH. Increase in EC_50_ occurred for GA/NADH/1.5 as well, but the decrease in the activity was not as significant as that for the GA/NADH/0.4 mixture. Like the trend in the EC_50_ values, a slight increase was observed in CI data calculated for GA/NADH/0.4 (0.92) and GA/NADH/1.5 (0.75) compared to the pH 7 results (Table S2). This increase means the synergistic effect weakened under such circumstances; however, it remains significant.

**Figure 4:**
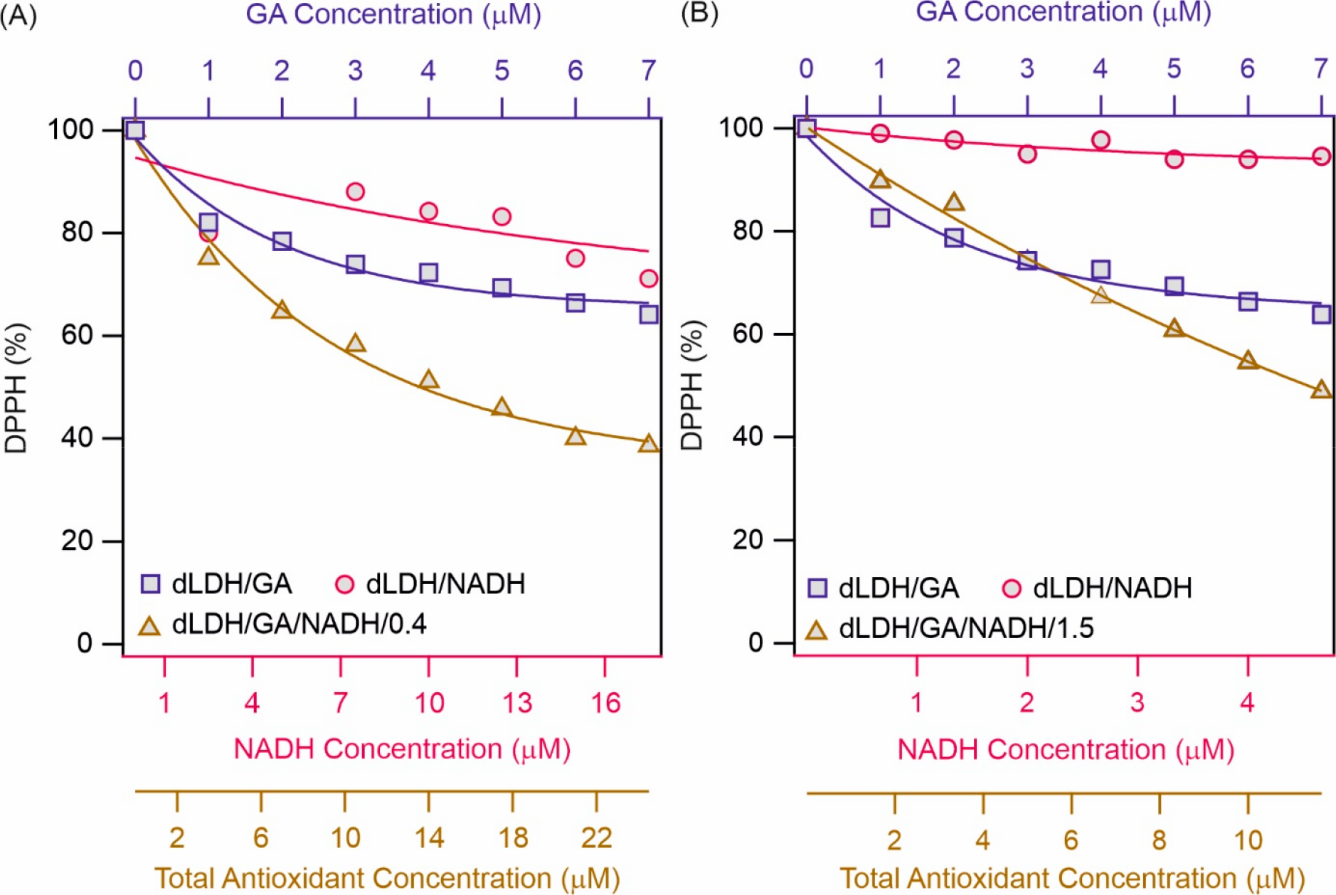
Radical scavenging activity of immobilized GA and NADH and coimmobilized at (A) 0.4 and (B) 1.5 molar ratios at pH 6. The lines are mathematical fits used to determine the EC_25_ values.

Upon immobilization of individual GA and NADH, their radical scavenging activity was limited, i.e., neither system reached the 50% DPPH (%) and thus, the EC_50_ values could not be determined for these composites (Figure S6). However, upon coimmobilization, both dLDH/GA/NADH/0.4 (Figure 4A) and dLDH/GA/NADH/1.5 (Figure 4B) reached 50% scavenging activity, and their EC_50_ values were 13.4 and 11.4 μM, respectively. In other words, once the antioxidants are immobilized together, the relatively high radical scavenging activity is kept due to the synergistic effect, while individual counterparts are rather inactive. Compared to the activity of the homogeneous GA/NADH/0.4 and GA/NADH/1.5 systems at pH 6, the EC_50_ values increased slightly, but they are still in the range to exhibit significant antioxidant effect by these composites.

Since dLDH/GA and dLDH/NADH did not reach 50% radical scavenging activity (Figure S6), the EC_25_ values (concentration needed to reach 25% DPPH decomposition) were used to calculate CI values. Calculation of the CI values for dLDH/GA/NADH/0.4 and dLDH/GA/NADH/1.5 revealed that at lower molar ratio, the interaction between the molecules remains similar; i.e., synergism occurred. However, at 1.5 molar ratio, the CI value became 1.49 indicating an antagonistic effect at 25% free radical scavenging stage (Table S2). Moreover, at higher antioxidant concentration, dLDH/GA/NADH/1.5 is able reach the 50% radical scavenging activity, while in contrast, the individually immobilized antioxidants are much less active. These facts clearly suggest that the combination of these molecules resulted in synergism even at 1.5 molar ratios once their concentration is higher than the one corresponds to the EC_50_ data.

To compare the radical scavenging activities with those of other systems, literature data are shown in Table S3. Those materials contained GA and other antioxidant molecules incorporated in various hybrid substances. Our composites, both dLDH/GA/NADH/0.4 and dLDH/GA/NADH/1.5, showed remarkable activity compared to the published data. Accordingly, the EC_50_ values determined in the present study are lower than the literature data, indicating the fact that combination of GA and NADH in a dLDH-based composite is a promising way to develop efficient antioxidant dispersions.

### Measurement of Copper Reducing Activity

**Figure 5:**
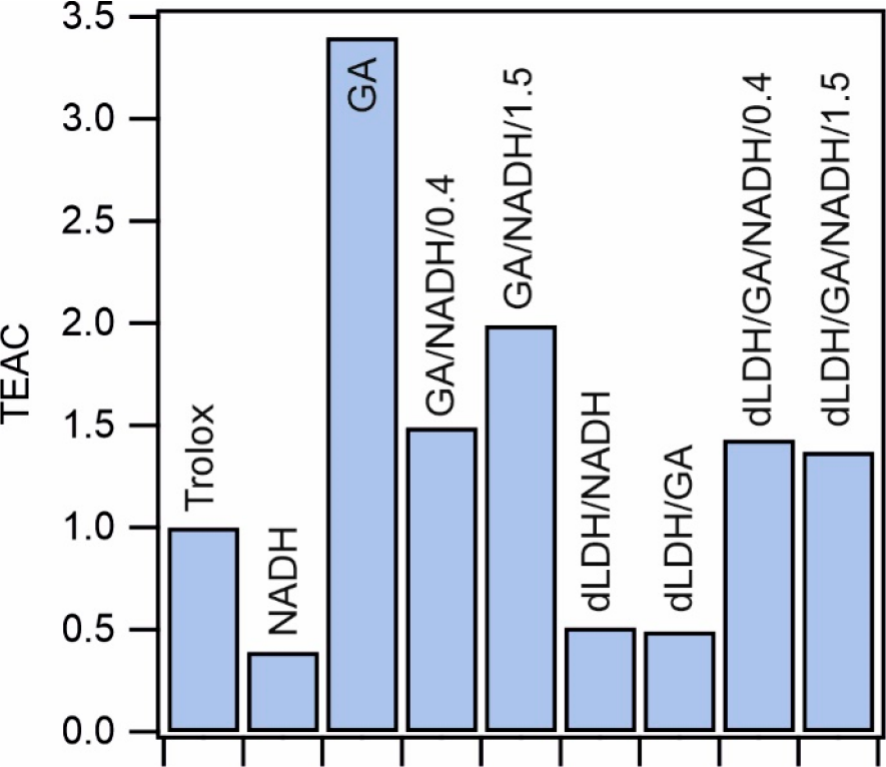
TEAC values of the antioxidants in dissolved and immobilized forms, including both individual and mixed samples.

To further evaluate the antioxidant activities, a CuPRAC assay was also performed. During the reaction, the Cu(II)(Nc)_2_ is reduced by the antioxidants and Cu(I)(Nc)_2_ is produced. By increasing the antioxidant concentration, the absorbance of the Cu(I)(Nc)_2_ complex at 450 nm increased (Figures S7 and S8). The activities are expressed in terms of TEAC values (Figure 5).

For the samples that contained solely one antioxidant, similar tendencies were discovered as in the DPPH assay discussed above. Accordingly, GA showed activity significantly higher than that of NADH in solution. Furthermore, the activity of the former one was more than three times higher than that for the reference trolox molecule. Unlike the results in the DPPH assay, the mixtures did not show higher activities than the antioxidants alone; therefore, the synergistic effect was not detected in homogeneous solutions by this method. However, one can note that both mixtures still showed remarkable copper reducing ability as the TEAC values were more than unity. Upon immobilization, a similar phenomenon occurred as that in the DPPH assay. If the antioxidants were immobilized individually, the activity decreased considerably; nevertheless, the simultaneous adsorption gave rise to an increased antioxidant power. The dLDH/GA/NADH/0.4 and dLDH/GA/NADH/1.5 composites possessed activities similar to those of GA/NADH/0.4 and GA/NADH/1.5. These results further prove that coimmobilization of GA and NADH is a suitable method to maintain considerable antioxidant activity compared to the immobilization of the sole molecules, during which the antioxidant efficiency limited or even lost due to the heterogenization procedure.

## Conclusions

The individual and joint antioxidant activities of a phenolic acid, GA, and an important biological cofactor, NADH, was determined. GA possesses an extraordinary free radical scavenging activity; however, NADH exhibited only limited antioxidant power in the tests. Their combination led to an increased antioxidant activity superior to the sum of the individual ones. Calculated CIs data revealed that the GA-NADH interaction was synergistic in the selected molar ratios. The molecular antioxidants were successfully immobilized by electrostatic interactions, while their coimmobilization was also performed on dLDH particles. The effect of adsorption on the surface charge features and particle aggregation processes was explored in light scattering measurements, while the colloidal stability regimes of the hybrid dispersions were unambiguously determined to select highly stable dispersions for further studies. The individually adsorbed antioxidants lost their radical scavenging activity; however, by coadsorbing them on dLDH at an optimized molar ratio, the synergistic antioxidant activity, which was discovered in homogeneous solutions, was maintained. The advantageous joint redox features of these molecules were further confirmed in the CuPRAC assay in both dissolved and immobilized forms, since similar tendencies were observed. Accordingly, considerable improvement was obtained in the activity of the coimmobilized substances compared to the case of individual adsorption suggesting the synergistic effect between the GA and NADH molecules. Beside enhancement of the GA activity upon adsorption on the dLDH surface, its radical scavenging ability was also improved in the presence of the NADH molecules. Our composites exhibited an antioxidant effect higher than that of the GA containing ones reported in the literature to date. Furthermore, the antioxidant activity of NADH was not investigated in detail earlier. In particular, the synergistic radical scavenging ability of heterogenized NADH in combination with GA is a very promising and novel finding. In conclusion, the simultaneous heterogenization of GA and NADH is a promising method to develop antioxidant materials of high colloidal stability and oxidative stress reducing power to be used in free radical or ROS scavenging in biomedical or industrial processes.

## Supporting Information Available

Results of antioxidant activity measurements, UV–vis spectra, zeta potential, absorbance and hydrodynamic radius data (PDF)
