# Supplementary material for: Molecular Antioxidants Maintain Synergistic Radical Scavenging Activity upon Co-Immobilization on Clay Nanoplatelets
Source: ACS Biomater Sci Eng. 2023 Sep 22;9(10):5622–31. doi: 10.1021/acsbiomaterials.3c00909 (PMC10565722; doi:10.1021/acsbiomaterials.3c00909)
Supplement: Supplementary file 1 — ab3c00909_si_001.pdf [file ab3c00909_si_001.pdf]

Supporting Information (SI)

Molecular Antioxidants Maintain Synergistic Radical Scavenging Activity upon Co-Immobilization on Clay Nanoplatelets

Adel Szerlauth, Szilárd Varga, Istvan Szilagyi*

MTA-SZTE Lendület Biocolloids Research Group, Department of Physical Chemistry and Materials Science, Interdisciplinary Excellence Centre, University of Szeged, H-6720 Szeged, Hungary

*Corresponding author. Email: szistvan@chem.u-szeged.hu

Table S1. EC_{50} and TEAC values of native and immobilized antioxidants.

Sample	EC_{50} (μM)	EC_{50} (pH 6)[a] (μM)	TEAC[b]
GA	6.0	5.8	3.40
NADH	22.6	56.4	0.39
GA/NADH/0.4[c]	8.8	14.8	1.49
GA/NADH/1.5[d]	5.5	6.7	1.99
dLDH/GA	N.A.	N.A.	0.49
dLDH/NADH	N.A.	N.A.	0.51
dLDH/GA/NADH/0.4	N.A.	13.4	1.43
dLDH/GA/NADH/1.5	N.A.	11.4	1.37

[a]EC_{50} values were calculated based on the measurements operated at pH 6. [b]TEAC means trolox equivalent antioxidant capacity, calculated by Eq. 6. [c]GA/NADH/0.4 means the mixture of GA and NADH in a molar ratio of 0.4. [d]GA/NADH/1.5 means the mixture of GA and NADH in a molar ratio of 1.5.

Table S2. CI values and type of interaction between the molecular antioxidants in solution and in the heterogenized forms.

Sample	CI[a]	Interaction
GA/NADH/0.4	0.70	Synergism
GA/NADH/0.4 (pH 6)	0.92	Synergism
GA/NADH/1.5	0.65	Synergism
GA/NADH/1.5 (pH 6)	0.75	Synergism
dLDH/GA/NADH/0.4	0.74	Synergism
dLDH/GA/NADH/1.5	1.49	Antagonism

[a]CI represents the combination index calculated by the Eq. 5.

Table S3. Antioxidant activity measured in DPPH assay for various antioxidant composites.

System	Antioxidant activity	Ref.
GA-LDH	Scavenging effect: ~93 %	1
GA-LDH	Scavenging ratio (870 min): 83.9 %	2
	IC_{50}:	
IONP@GA1[a]	2.7 mg/mL	3
IONP@GA2	2.2 mg/mL	
IONP@GA3	1 mg/mL	
GA + resveratrol	IC_{50} (30 min): 922.5 □M	4
CG		
CG/PVA/AgNPs-0	scavenging activity (%)	
CG/PVA/AgNPs-1	~ 80 %	5
CG/PVA/AgNPs-2		
CG/PVA/AgNPs-3		
	EC_{50}:	
LDH/TA/a	~ 0.2 µM	
LDH/GSH/a	~ 500 µM	6
LDH/GSH/c	~ 600 µM	
	EC_{50}:	
EA/LDH	87.3 µM	
MeOH-EA-LDH	41.9 µM	
EtOH-EA-LDH	19.8 µM	7
AC-EA-LDH	21.7 µM	
CAN-EA-LDH	16.1 µM	
DMF-EA-LDH	30.1 µM	

[a]IONP represents Fe_3O_4 nanoparticles, while the numbers refer to the increasing particle size.

[b]CG represents gallic acid grafted chitosan film, while CG/PVA/AgNPs-0-3 refers to composite of gallic acid grafted chitosan, poly(vinyl-alcohol) and silver nanoparticle.

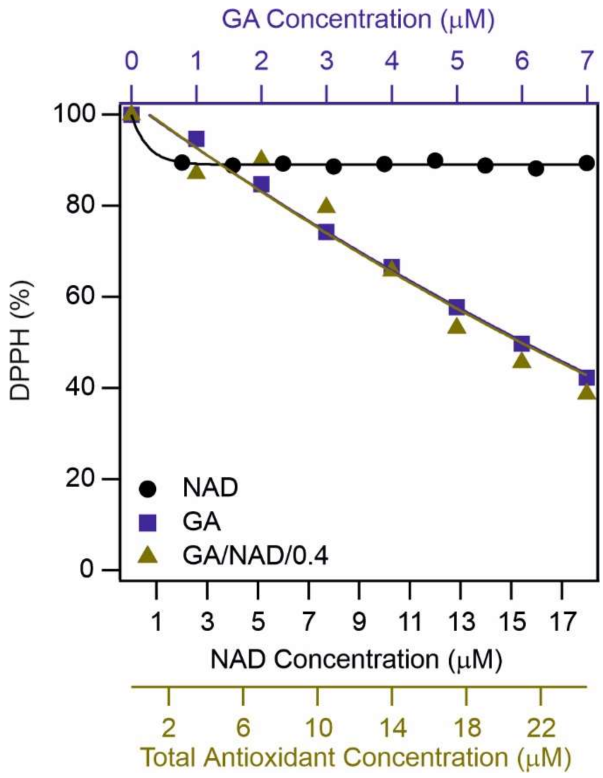

Figure S1. Free radical scavenging activity of GA, NAD, and their combination.

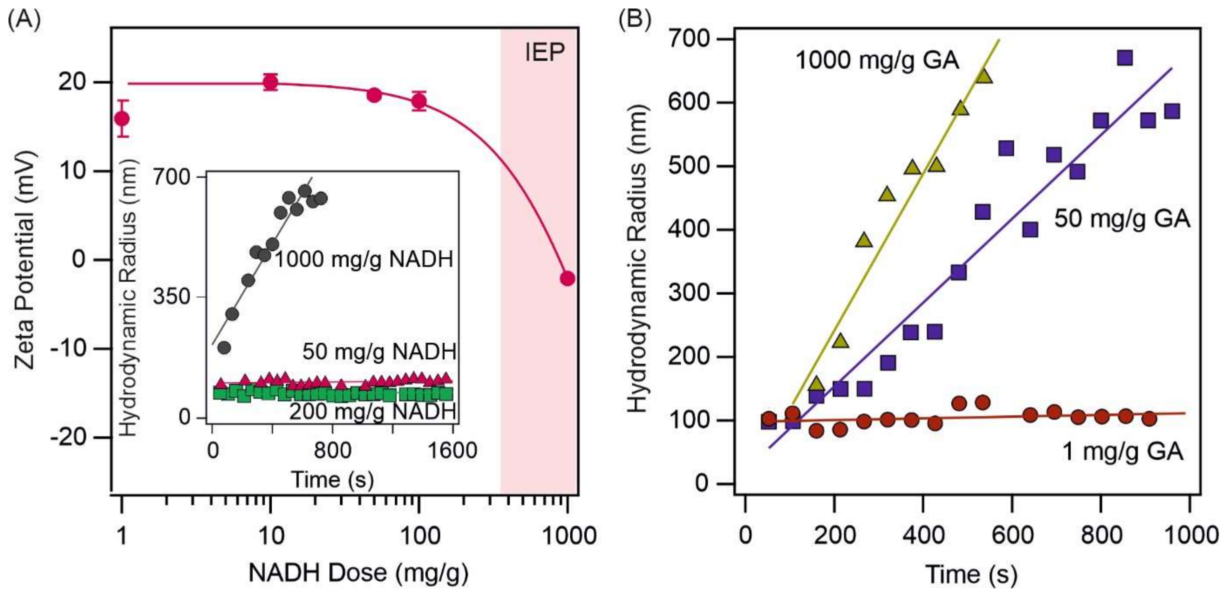

Figure S2. (A) Zeta potential of dLDH versus NADH dose. The inset shows time-resolved hydrodynamic radius data at different NADH concentrations. (B) Hydrodynamic radii values measured at different time intervals for dLDH particles in the presence of GA of various concentrations. During these measurements, the pH was set to 7 and 1 mM NaCl concentration was applied as a background electrolyte.

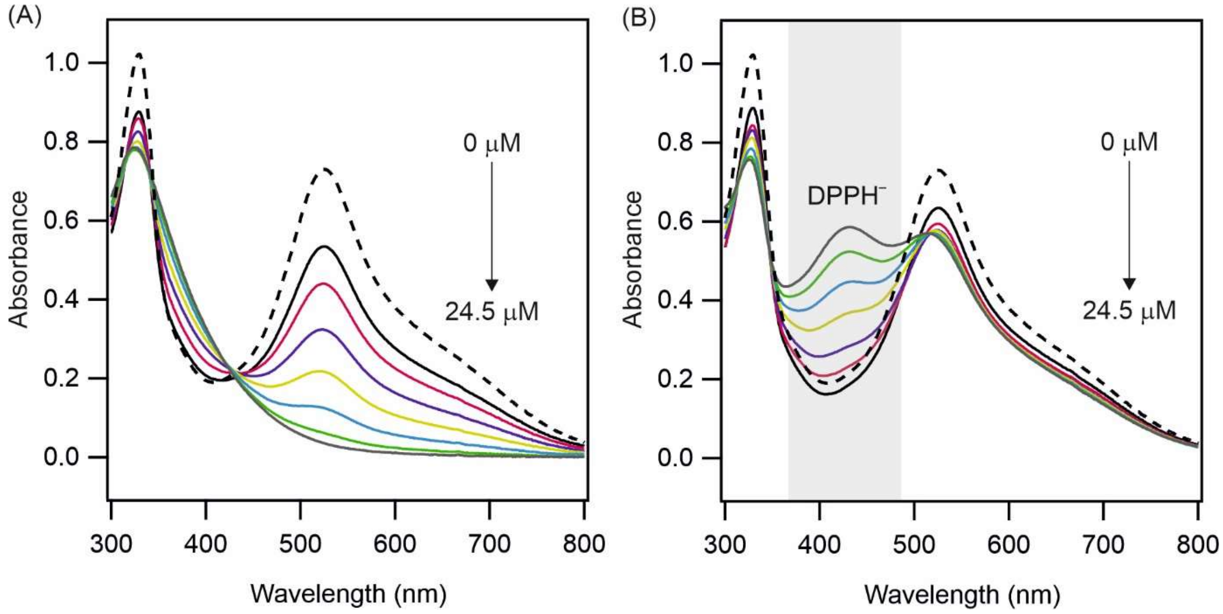

Figure S3. UV-VIS spectra of DPPH samples after addition of (A) GA/NADH/0.4 mixed solutions and (B) immobilized antioxidant (dLDH/GA/NADH/0.4) dispersions in different concentrations. The total antioxidant concentration was varied in 0 – 24.5 μM range. The location of the characteristic peak of the $DPPH^-$ anion is labelled in (B) with the grey area. The dashed spectrum represent the DPPH absorption spectrum without any additional antioxidant solution.

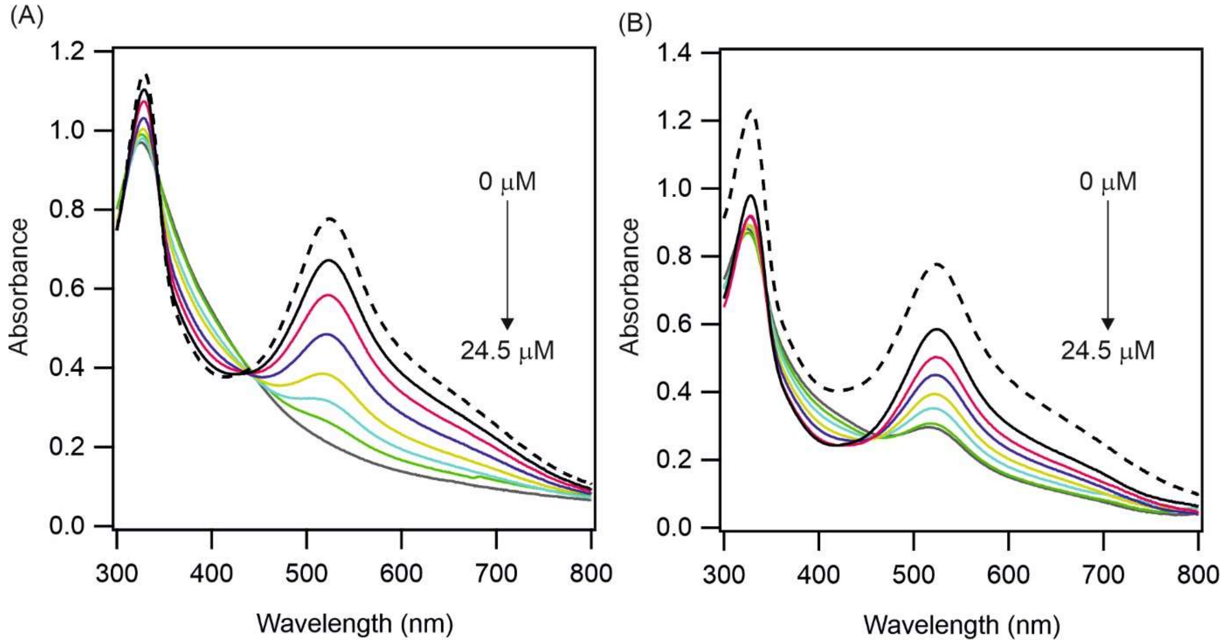

Figure S4. Spectra of DPPH solution at pH 6 buffer after addition of (A) GA/NADH/0.4 mixed solutions and (B) immobilized antioxidant (dLDH/GA/NADH/0.4) dispersions in different concentrations. The total antioxidant concentration was varied in 0 – 24.5 μM range. The dashed spectrum represent the DPPH absorption spectrum without any additional antioxidant solution.

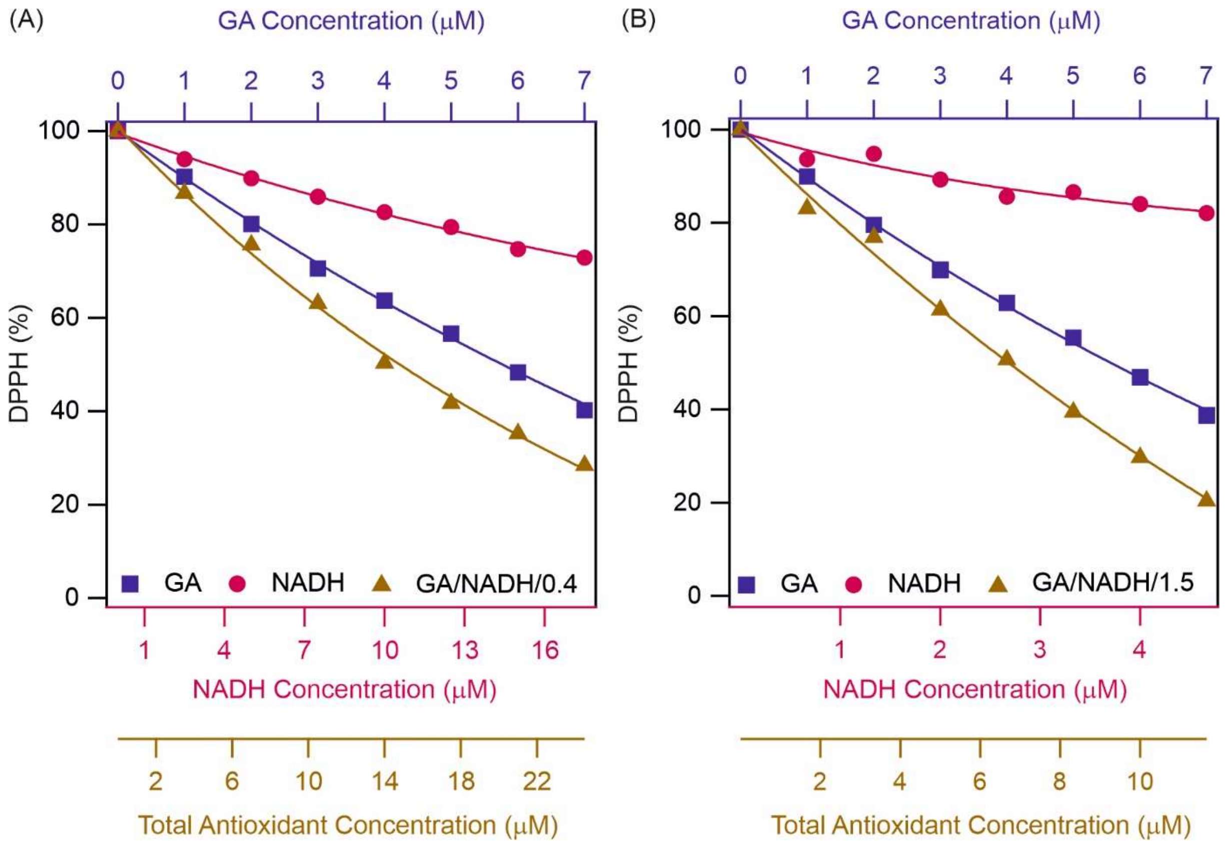

Figure S5. Radical scavenging activity of the antioxidants alone and the mixtures at pH 6 at 0.4 (A) and 1.5 (B) GA/NADH molar ratio. The lines are mathematical fits used to determine the EC_{50} values.

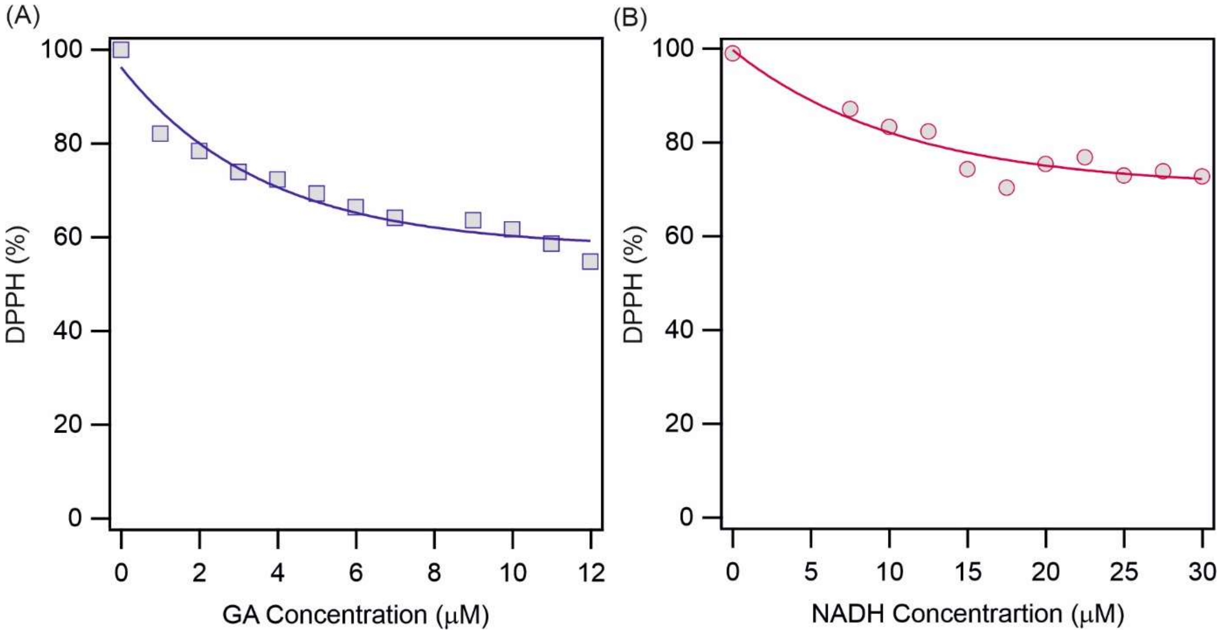

Figure S6. DPPH assay for dLDH/GA (A) and dLDH/NADH (B) in an extended antioxidant concentration range.

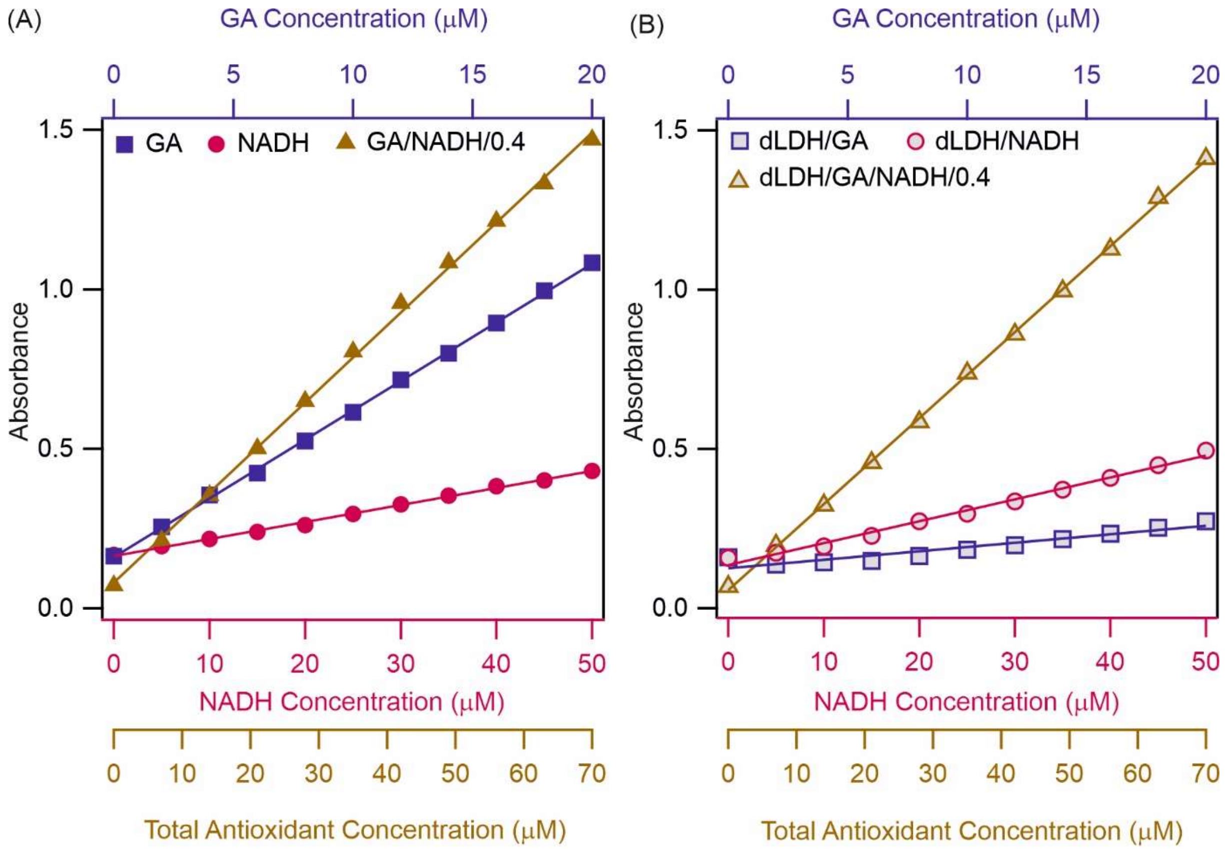

Figure S7. Absorbance versus antioxidant concentration for the antioxidants (A) in solution and (B) in immobilized form. The molar GA/NADH ratio was 0.4 in the mixed samples.

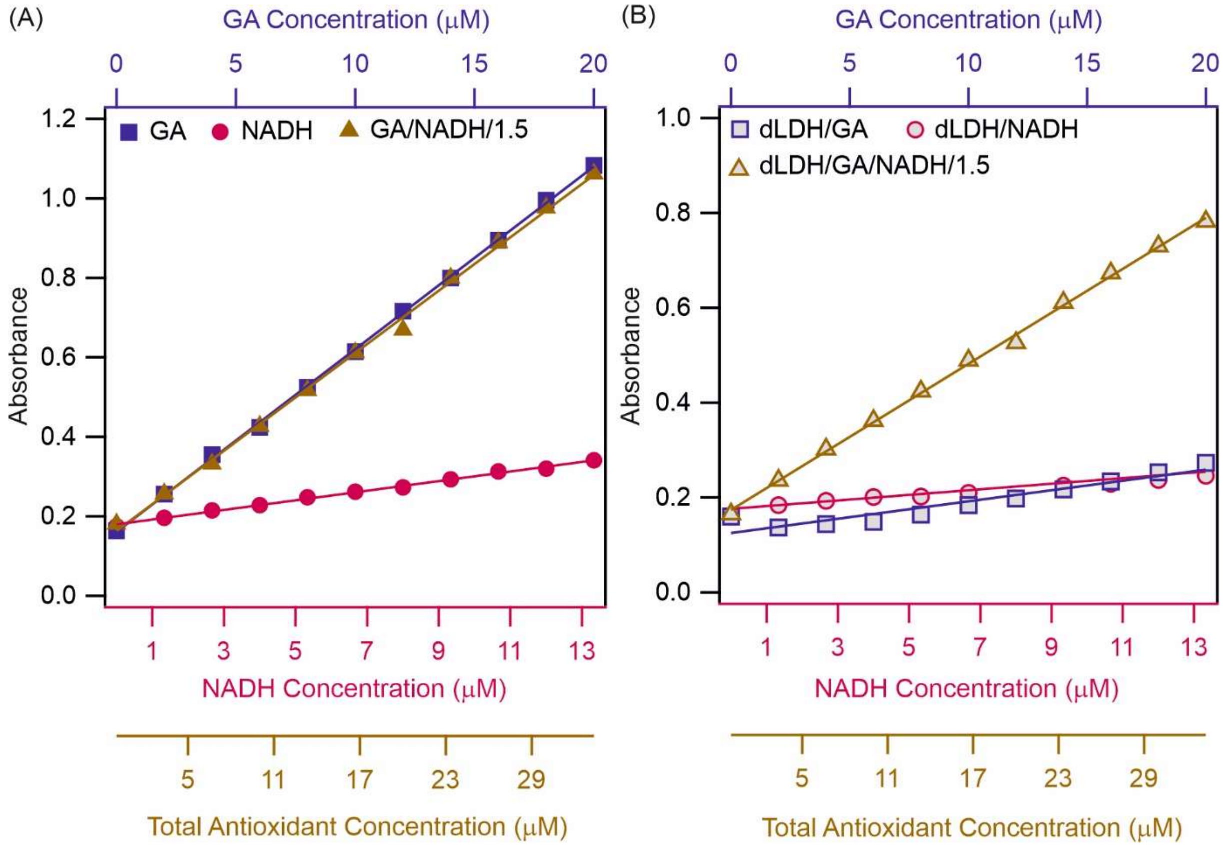

Figure S8. Absorbance versus antioxidant concentration for the antioxidants (A) in solution and (B) in immobilized form. The molar GA/NADH ratio was 1.5 in the mixed samples.

REFERENCES

(1) Ansy, K. M.; Lee, J. H.; Piao, H.; Choi, G.; Choy, J. H. Stabilization of antioxidant gallate in layered double hydroxide by exfoliation and reassembling reaction. *Solid State Sci.* **2018**, *80*, 65-71.

(2) Kong, X. G.; Jin, L.; Wei, M.; Duan, X. Antioxidant drugs intercalated into layered double hydroxide: Structure and in vitro release. *Appl. Clay Sci.* **2010**, *49*, 324-329.

(3) Shah, S. T.; Yehye, W. A.; Saad, O.; Simarani, K.; Chowdhury, Z. Z.; Alhadi, A. A.; Al-Ani, L. A.; Surface functionalization of iron oxide nanoparticles with gallic acid as potential antioxidant and antimicrobial agents. *Nanomaterials* **2017**, *7*, 306.

(4) Skroza, D.; Mekinic, I. G.; Svilovic, S.; Simat, V.; Katalinic, V. Investigation of the potential synergistic effect of resveratrol with other phenolic compounds: A case of binary phenolic mixtures. *J. Food Compos. Anal.* **2015**, *38*, 13-18.

(5) Zhao, Y.; Yang, L.; Xu, M.; Wang, H.; Gao, X.; Niu, B.; Li, W. Gallic acid functionalized chitosan immobilized nanosilver for modified chitosan/Poly (vinyl alcohol) composite film. *Int. J. Biol. Macromol.* **2022**, *222*, 2987-3000.

(6) Szerlauth, A.; Kónya, Z. D.; Papp, G.; Kónya, Z.; Kukovecz, Á.; Szabados, M.; Varga, G.; Szilágyi, I. Molecular orientation rules the efficiency of immobilized antioxidants. *J. Colloid Interface Sci.* **2023**, *632*, 260-270.

(7) Murath, S.; Szerlauth, S.; Sebok, D.; Szilagyi, I. Layered double hydroxide nanoparticles to overcome the hydrophobicity of ellagic acid: An antioxidant hybrid material. *Antioxidants* **2020**, *9*, 153.
